# Supplementary material for: Shared somatosensory–motor neural population dynamics track motor recovery after stroke
Source: bioRxiv. 2026 Feb 12:2026.02.10.705199. Preprint. [Version 1] doi: 10.64898/2026.02.10.705199 (PMC12919233; doi:10.64898/2026.02.10.705199)
Supplement: Supplement 1 [file NIHPP2026.02.10.705199v1-supplement-1.pdf]

bioRxiv preprint doi: https://doi.org/10.64898/2026.02.10.705199; this version posted February 12, 2026. The copyright holder for this preprint (which was not certified by peer review) is the author/funder, who has granted bioRxiv a license to display the preprint in perpetuity. It is made available under aCC-BY 4.0 International license.

SUPPLEMENTAL FIGURES

SFig 1: Decoding parameter search

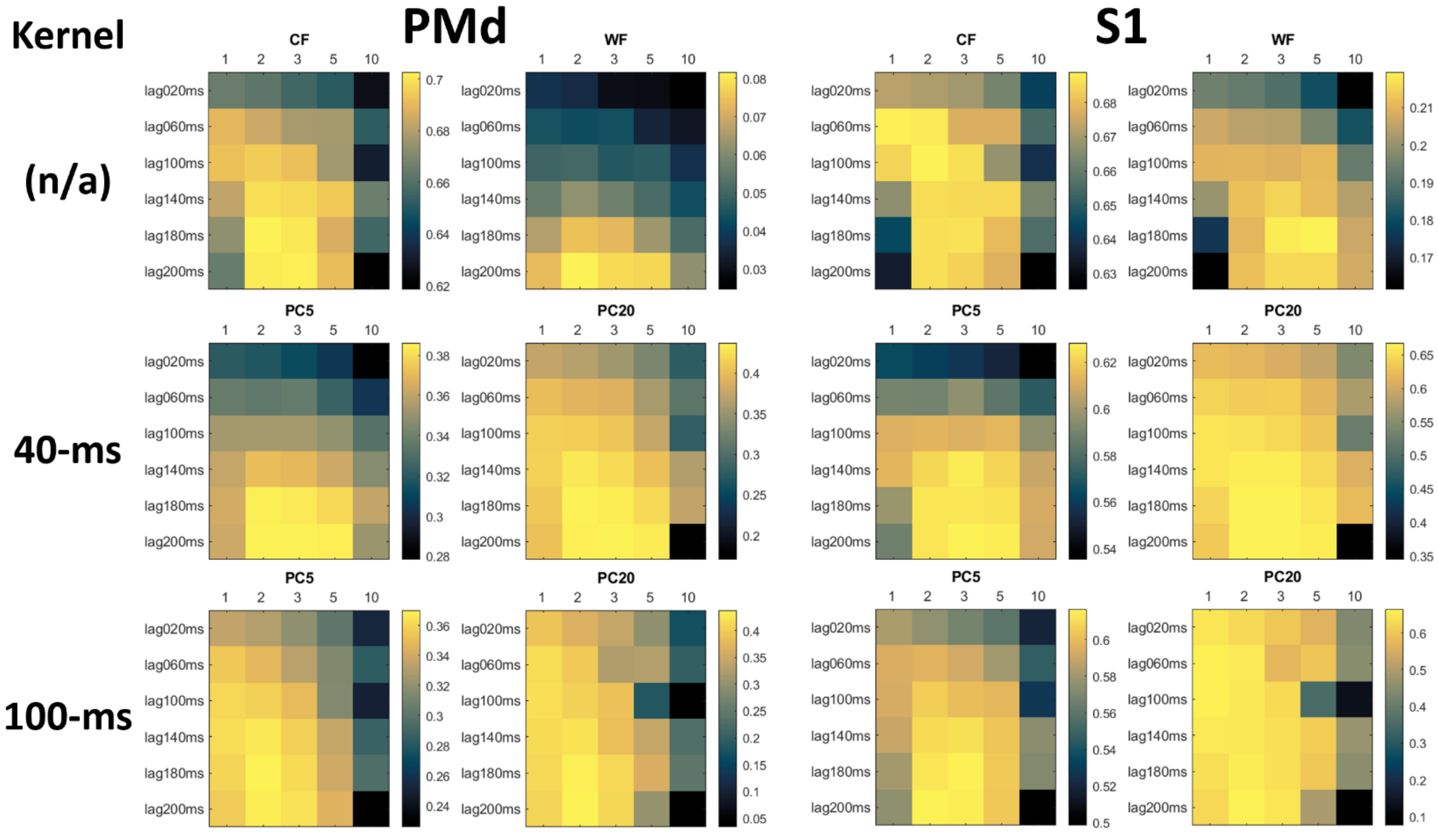

Supplemental Figure 1. Decoding parameter search

bioRxiv preprint doi: https://doi.org/10.64898/2026.02.10.705199; this version posted February 12, 2026. The copyright holder for this preprint (which was not certified by peer review) is the author/funder, who has granted bioRxiv a license to display the preprint in perpetuity. It is made available under aCC-BY 4.0 International license.

SFig 2: Recovery: WFs

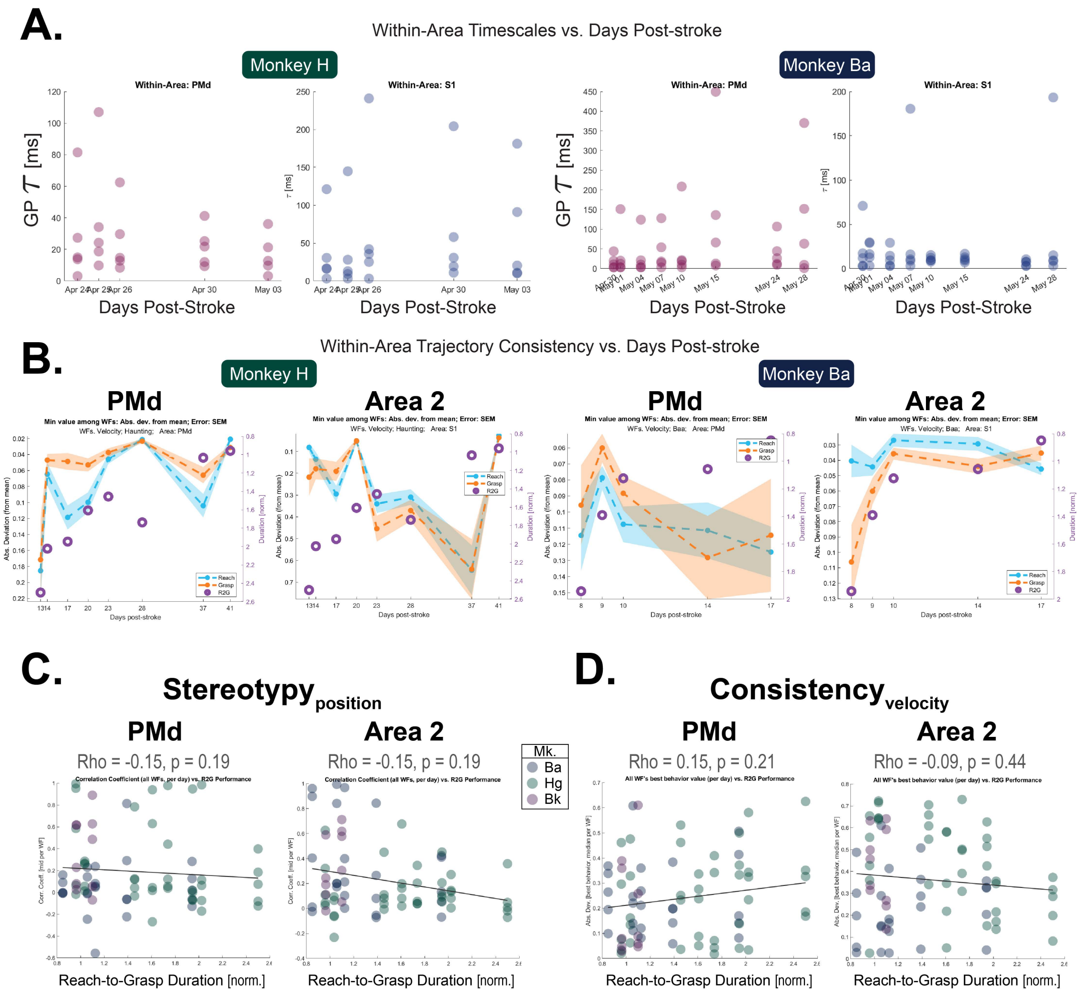

*Supplemental Figure 2. **Within-area factors did not reliably track neural recovery.***

WF results for the same two animals that showed post-lesion behavioral deficits (n = 2) as shown in Figure 4 and Figure 5. All DLAG results in this figure are from models trained on low-SBP data. Values are separated per area (PMd and Area 2) for all tiles.

(A) WF Gaussian process widths (τ) per session over time. Left two tiles: Mk. H; Right two tiles: Mk. Ba.

(B) WF trajectory consistency (cyan and orange) and R2G performance (purple). Showing results for the most consistent WF for two R2G timepoint subsets: reach start (cyan) and grasp (orange); "grasp" is the mean of grasp start and grasp finish. Midlines: median per-trial consistency. Error: SEM. Normalized R2G duration per session (purple).

(C) WF trajectory stereotypy$_{position}$ vs. normalized R2G duration for all animals (n = 3). Per area, the three most stereotyped WFs for each session are included. Line shows linear model fit.

(D) WF instantaneous consistency$_{velocity}$ vs. normalized R2G duration for all animals (n = 3). Per area, the three most consistent WFs for each session are included. Line shows linear model fit.
